# A novel γD-crystallin mutation causes mild changes in protein properties but leads to congenital coralliform cataract

**Published:** 2009-08-06

**Authors:** Li-Yun Zhang, Bo Gong, Jian-Ping Tong, Dorothy Shu-Ping Fan, Sylvia Wai-Yee Chiang, Dinghua Lou, Dennis Shun-Chiu Lam, Gary Hin-Fai Yam, Chi-Pui Pang

**Affiliations:** 1 Department of Ophthalmology and Visual Sciences, The Chinese University of Hong Kong, Hong Kong, China; 2 Department of Ophthalmology, the First Affiliated Hospital, College of Medicine, Zhejiang University, Hangzhou, China

## Abstract

**Purpose:**

To identify the genetic lesions for congenital coralliform cataract.

**Methods:**

Two Chinese families with autosomal dominant coralliform cataract, 12 affected and 14 unaffected individuals, were recruited. Fifteen known genes associated with autosomal dominant congenital cataract were screened by two-point linkage analysis with gene based single nucleotide polymorphisms and microsatellite markers. Sequence variations were identified. Recombinant FLAG-tagged wild type or mutant γD-crystallin was expressed in human lens epithelial cells and COS-7 cells. Protein solubility and intracellular distribution were analyzed by western blotting and immunofluorescence, respectively.

**Results:**

A novel heterozygous change, c.43C>A (R15S) of γD-crystallin (*CRYGD*) co-segregated with coralliform cataract in one family and a known substitution, c.70C>A (P24T), in the other family. Unaffected family members and 103 unrelated control subjects did not carry these mutations. Similar to the wild type protein, R15S γD-crystallin was detergent soluble and was located in the cytoplasm. ProtScale and ScanProsite analyses revealed raised local hydrophobicity and the creation of a hypothetical casein kinase II phosphorylation site.

**Conclusions:**

A novel R15S mutation caused congenital coralliform cataract in a Chinese family. R15S possessed similar properties to the wild type γD-crystallin, but its predicted increase of hydrophobicity and putative phosphorylation site could lead to protein aggregation, subsequently causing opacification in lens.

## Introduction

Congenital cataract refers to lens opacification presented at birth or developed shortly thereafter. Its prevalence is up to 7.2 per 10,000 live births and renders about 10% of childhood blindness worldwide [1-7]. If left untreated, permanent visual loss usually occurs. Various etiological factors have been identified including infection, metabolic disorders, and genetic defects. About 18% of affected children have known family history of cataract [8]. The most common mode of genetic lesion is a single gene determinant in Mendelian inheritance. Autosomal dominant congenital cataract (ADCC) is a major form. Autosomal recessive and X-linked inheritance also exists [9]. According to the outward appearance, size, and location of lens opacity, congenital cataract (CC) is classified into various subtypes: whole lens, nuclear, lamellar, cortical, polar, sutural, pulverulent, cerulean, coralliform, and other minor subtypes [10,11]. The development of each type of cataract can be caused by distinctive etiological factors, especially defects in lens crystallins [9].

More than 20 genes out of 34 genetic loci mapped for isolated congenital cataract have been identified with specific mutations [12]. More than half of CC families carry mutations in 10 crystallin genes (namely crystallin alpha A (*CRYAA*),**crystallin alpha B (*CRYAB*), crystallin beta B1 (*CRYBB1*), crystallin beta B2 (*CRYBB2*), crystallin beta B3 (*CRYBB3*), crystallin beta A1**(*CRYBA1*), crystallin beta A4 (*CRYBA4*), crystallin gamma C**(*CRYGC*)*, *crystallin gamma D**(*CRYGD*) and crystallin gamma S**(*CRYGS*). About 25% of affected families have gene defects in membrane transport genes (major intrinsic protein of lens fiber (*MIP*)*,* gap junction protein alpha 8**(*GJA8*)*, *gap junction protein, alpha 3**(*GJA3*) and transmembrane protein 114**(*TMEM114*)) [13] and lens intrinsic membrane protein 2**(*LIM2*). The remaining are caused by mutations in genes encoding cytoskeletal proteins (beaded filament structural protein 1**(*BFSP1*) [14] and beaded filament structural protein 2**(*BFSP2*)), transcription factors (paired-like homeodomain 3**(*PITX3*)*,* v-maf musculoaponeurotic fibrosarcoma oncogene homolog**(*MAF*)*,* and heat shock transcription factor 4**(*HSF4*)), chromatin modifying protein (*CHMP4B*) [15], and glucosaminyl transferase 2 (*GCNT2*) [16]. Forkhead box E3 *(FOXE3*)*,* eyes absent homolog 1**(*EYA1*)*,* and paired box gene 6**(*PAX6*) have been reported to cause congenital cataract in some patients associated with other anterior segment anomalies [9]. The same mutation in different families or even within a family can result in drastically different morphologies and severity of lens opacification. On the other hand, similar or identical cataract presentation may arise from mutations of different genes. These observations suggest that additional genes or modifying factors such as environmental regulators could play important roles in cataract onset, progression, and maturation.

CRYGD is a structural protein essential for lens transparency. Mutations of *CRYGD* are common genetic lesions causing different types of congenital cataracts. Among the reported families with congenital cataract caused by mutations of crystallin, one-third of them were associated with *CRYGD*. Until now, a total of 11 cataract-causing mutations (UniProt) have been reported including R15C, P24S, P24T, R37S, R59H, G61C, E107A, Y134X, W156X, G165fsX8, and R168W [17-21]. In this study, we added a novel R15S mutation to this list, affirming a causative role of *CRYGD* in coralliform type of congenital cataract.

## Methods

### Patients and controls

**Figure 1:**
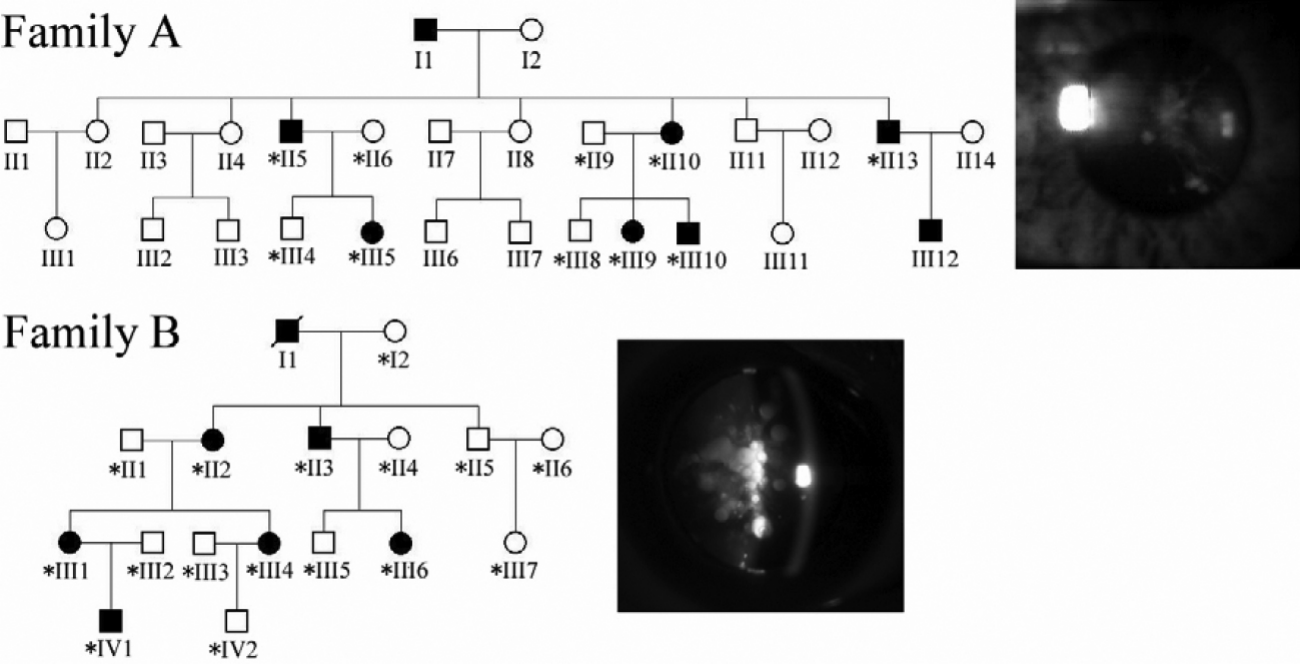
Pedigree of coralliform cataract families. The asterisk indicates family members who attended this study. The lens photograph from II-13 in Family A showed a line-shape opacity with a larger white dot end and tubular opacity radiating from the center of the lens. The lens photograph from II-2 in Family B showed a tubular and irregular opacity extending from center of the lens. Large white dots accumulated at the end of tubular protrusions.

This study adhered to the tenets of the Declaration of Helsinki and was approved by the ethics committees for medical research at The Chinese University of Hong Kong (Hong Kong, China) and Zhejiang University (Hangzhou, China). Two Chinese families with autosomal dominant congenital cataract were recruited at the University Eye Center (The Chinese University of Hong Kong; Family A; Figure 1) and the Department of Ophthalmology at the First Affiliated Hospital (College of Medicine at Zhejiang University; Family B; Figure 1). A total of 26 family members including 12 affected and 14 unaffected individuals attended this study, and informed consents were obtained from all participants. Unrelated Chinese control subjects (n=103) attending the hospital clinics for ophthalmic examinations were also recruited. They did not have any eye diseases except senile cataract and mild floaters. All subjects underwent complete ophthalmoscopic examinations. Family history and ophthalmic examination were documented by senior ophthalmologists. Peripheral venous blood was collected for genomic DNA extraction using QIAamp DNA kit (Qiagen, Valencia, CA).

### Candidate gene screening

Fifteen candidate genes that account for most cases of ADCC were taken for linkage analysis (Table 1). A gene exclusion strategy was conducted by screening with single nucleotide polymorphisms (SNPs) and microsatellite markers. We obtained gene-based SNP markers in Han Chinese from HapMap with ABI SNP browser v.3.5 (Applied Biosystems, Foster, CA). TaqMan SNP genotyping assay and allelic discrimination was conducted on an ABI PRISM 7000 sequence detection system (Applied Biosystems). For genes without informative SNP markers or those that could not be excluded by SNP linkage analysis, microsatellite markers flanking to the target genes were chosen from the Marshfield genetic map. GeneScan was conducted on ABIPRISM^®^ 377 DNA sequencer (Applied Biosystems). The pedigree and genotyping data were managed by GenoPedigree 1.0 and GeneBase 2.0.1. (Applied Biosystems). Two point LOD scores were calculated by the MLINK subprogram of FASTLINKAGE v.4.1P. A gene frequency of 0.0001 and penetrance of 100% were assumed for ADCC.

**Table 1:** SNPs and microsatellite markers selected in 15 known ADCC candidate genes for the linkage analysis.

**Gene**	**Gene ID**	**Chromosome location**	**SNPs**	**STRs**
*CRYAA*	1409	21q22.3	rs870137	D21S1890
				D21S266
				D21S1255
*CRYAB*	1410	11q22–22.3	rs2070894	D11S1986
			rs1940392	D11S4078
			rs14133	D11S1793
			rs10502149	D11S4151
*CRYBB1*	1414	22q11	rs4822752	
			rs5752351	
			rs2071859	
			rs4822749	
			rs5761618	
*CRYBB2*	1415	22q11.2	rs739315	D22S315
			rs5752084	D22S1174
			rs969623	
*CRYBA1*	1411	17q11.1–12	rs8080081	
*CRYGC*	1420	2q33–35	rs2242071	D2S2208
				D2S2361
*CRYGD*	1421	2q33–35	rs2305429	
*GRYGS*	1427	3q25-qter	rs3774803	D3S1262
			rs11917060	D3S3570
			rs1447670	
			rs4686428	
*MIP*	4284	12q12	rs7953824	D12S1632
			rs2269348	D12S1691
			rs3809125	
			rs10876890	
*GJA8*	2703	1q21–25	rs7541950	D1S498
			rs2132397	D1S442
*GJA3*	2700	13q11–13	rs4769953	
			rs1886176	
*BFSP2*	8419	3q21–22	rs1153876	D3S1290
			rs666067	D3S3713
			rs4854585	D3S3657
			rs6762405	D3S1292
			rs931099	
*PITX3*	5309	10q24–25	rs3758553	D10S1267
*MAF*	4094	16q23.2	rs2288066	D16S3040
				D16S504
				D16S503
*HSF4*	3299	16q22	Hcv25749941	
			Hcv25613880	
			rs11642409	
			rs9033	

Gene name, gene ID in NCBI GeneBank, and the chromosomal location for 15 candidate genes were shown in this table. All single nucleotide polymorphism (SNP) markers and microsatellite markers used in this study were listed, respectively.

### Mutation analysis

All coding exons and splice sites of *CRYAA*, *CRYAB*, *CRYGC*, *CRYGD*, and *MAF *were sequenced using BigDye terminator v3.1 cycle sequencing kit (Applied Biosystems) and specific primers (Table 2) and detected by ABIPRISM^TM^ 377 DNA sequencer. The data were compared with sequences from NCBI GenBank (*CRYAA*: NM_000394, *CRYAB*: NG_009824; *CRYGC*: NM_020989; *CRYGD*: NM_006891.3; *MAF*: NM_001031804).

**Table 2:** Specific primers for direct sequencing.

**Gene**	**Amplicon**	**Primer sequences**
*CRYAA*	1	5′-CTCCAGGTCCCCGTGGTA
		5′-AGGAGAGGCCAGCACCAC
	2	5′-CTGTCTCTGCCAACCCCAG
		5′-CTGTCCCACCTCTCAGTGCC
	3	5′-AATGATCCTGCGATTTTGGAG
		5′-GGAAGCAAAGGAAGACAGACACC
*CRYAB*	1	5′-TGTAGCTGCAGCTGAAGGAG
		5′-TTCCAGTAAGGACTCTCCCG
	2	5′-GAAGGATGAATTACCCGGACAG
		5′-AGACATTGATTTGTAACCCCTGATC
	3	5′-GAGTTCTGGGCAGGTGATAAT
		5′-CTGGTGGGGAAACTTTCTTG
*CRYGC*	1	5′-TGCATAAAATCCCCTTACCGC
		5′-ACTCTGGCGGCATGATGG
	2	5′-AGACTCATTTGCTTTTTTCCATCC
		5′-GAATGACAGAAGTCAGCAATTGC
*CRYGD*	1	5′-CAGCAGCCCTCCTGCTAT
		5′-GCTTATGTGGGGAGCAAACT
	2	5′-CTTTTCTTCTCTTTTTATTTCTGGGTCC
		5′-GAAAGACACAAGCAAATCAGTGCC
*MAF*	1	5′-CTCCTGCAGCCCATCTGG
		5′-CTGGTGGCTGTTGCTGATG
	2	5′-CATCAGCAACAGCCACCAG
		5′-GAGAAGCGGTCGTCGAAGT
	3	5′-ACTTCGACGACCGCTTCTC
		5′-TGGCGAGCATGGCTCTAG
	4	5′-CCTTTACGCTGCGTTTGATC
		5′-AACCCCCAGACAAGAGGC

Five genes which were sequenced were listed. Forward and reverse primer sequences were provided for each amplicon of each gene.

### Computational analysis

Effects of amino acid changes on the CRYGD protein structure, the isoelectric point (pI), and molecular weight (MW) were examined by Expasy proteomics. Local hydrophobicity was predicted by ProtScale. The protein sequence was scanned by ScanProsite to predict the effect of the mutation on specific motifs.

### *CRYGD* expression, mutagenesis, and transfection

Human full-length wild type *CRYGD* was cloned to p3XFLAG-myc-CMV^™^-25 (Sigma, St Louis, MO) for epitope tagging to generate pFLAG/myc-CRYGD^WT^ [19]. Missense mutations were introduced by a site-directed mutagenesis kit (Stratagene, La Jolla, CA) with specific oligonucleotides (Table 3). Correctness of the construct sequence was confirmed by direct sequencing. Preparation of pFLAG-CRYGD^G165fs^ was described as before [19]. Human lens B3 epithelial cells and COS-7 cells (ATCC, Manassas, VA) were maintained in Eagle’s minimum essential medium (Invitrogen, Carlsbad, CA) supplemented with 10% fetal bovine serum and antibiotics [19]. Cells were seeded with 5×10^5^ cells in a 60 mm (diameter) culture dish (Nunc, Rochester, NY) overnight before transfection. Expression construct containing wild type or mutant *CRYGD* was mixed with FuGene HD reagent (Roche, Basel, Switzerland) at a ratio of 3 μl of FuGene per 1 μg of vector DNA in Opti-MEM® I (Invitrogen) supplemented with GlutaMAX™-I (Invitrogen) and incubated for 30 min. The mixture was then added to cells for up to 48 h.

**Table 3:** Sense oligonucleotides for site-directed mutagenesis in *CRYGD*.

**Mutations**	**Oligonucleotides with specific base change (underlined)**
R15C	5′-GACCGGGGCTTCCAGGGCTGCCACTATGAATGCAGC
R15S	5′-GACCGGGGCTTCCAGGGCAGCCACTATGAATGCAGC
P24T	5′-GAATGCAGCAGCGACCACACCAACCTGCAGCCCTAC
G61C	5′-TACTTCCTGCGCCGCTGCGACTATGCCGAC

Constructs of four mutations in *CRYGD* were made. The sense oligonucleotides used in site-directed mutagenesis experiment were listed, respectively.

### Detergent solubility of wild type and mutant CRYGD

Cells transiently expressing wild type or mutant CRYGD were washed twice with ice-cold PBS and lysed in 2.5×10^6^ cells/ml lysis buffer, which contained 0.5% Triton X-100 (Tx; Sigma), for 2 min on ice [22]. After centrifugation, the Tx-soluble fraction was collected and denatured in SDS buffer containing 50 mM DTT. The pellet containing Tx-insoluble proteins was sonicated and denatured in SDS buffer containing 9 M urea. Both Tx-soluble and Tx-insoluble proteins equivalent to 7.5×10^4^ cells were analyzed by western blotting using monoclonal antibodies against FLAG, Glyceraldehyde 3-phosphate dehydrogenase (GAPDH), or β-actin (Sigma).

### Immunofluorescence staining

Cells grown on glass coverslips were fixed with freshly prepared neutral buffered 2% paraformaldehyde (Sigma) and permeabilized with 0.05% Tx [22] followed by incubations with mouse monoclonal antibodies against FLAG or mouse monoclonal antibodies against CRYGD (Abnova, Heidelberg, Germany). Rhodamine Red-X goat anti-mouse IgG (Invitrogen) was applied as the secondary antibody. The nuclei were contrast-stained with 4',6-diamidino-2-phenylindole (DAPI). The cells were examined under fluorescence microscopy (DMRB, Leica, Germany), which was equipped with Spot RT color system (Diagnostic Instruments Inc., Sterling Heights, MI).

## Results

### Clinical investigations

Two pedigrees exhibited coralliform type of cataract in an autosomal dominant mode of inheritance. All affected patients had bilateral lens opacification, characterized by the appearance of white lines and processes extending from the nucleus to peripheral cortex, resembling the shape of sea coral (Figure 1). This was classified as the coralliform type of cataract by senior ophthalmologists in two eye centers (D.S.P.F., D.L., and J.P.T.). The lens opacity was less severe in terms of size and density in Family A than in Family B and did not result in significant loss of visual acuity (VA). In family A, except for one patient, II10, who had low vision due to high myopia, VA of other patients ranged from 0.67 to 0.8 without lens surgery. Patient III-10 was recorded to have normal lens transparency at his first eye examination at the age of 2.5 years, but he was diagnosed to have cataract at 10 years old. Other family members were diagnosed after the age of five years. In Family B, all patients showed cataract within the first year after birth. The lens opacity caused obvious vision loss ranging from 0.04 to 0.7. Four of the six patients received cataract surgeries before the age of 30.

### Linkage analysis and DNA sequencing

**Figure 2:**
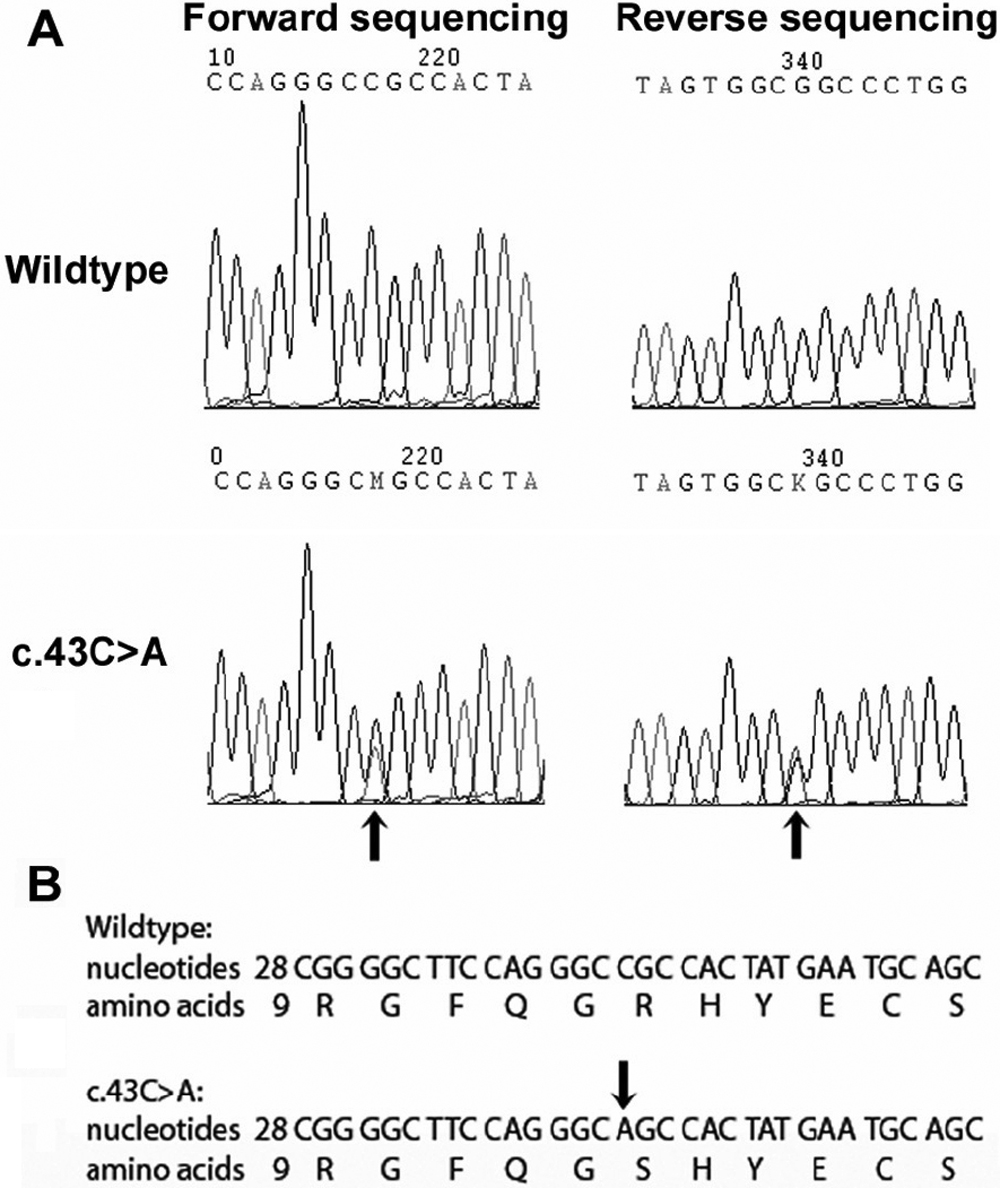
R15S mutation in Family A. **A**: The upper panel showed the forward (on the left) and the reverse (on the right) DNA segments of wildtype *CRYGD*. The lower panel displayed the forward and the reverse sequences of mutant *CRYGD*. The mutation of c.43C>A was indicated by arrow both in forward and reverse sequences. **B**: The DNA sequences in the upper lines and amino acid sequences in the lower lines were compared between wildtype and mutant *CRYGD.* The arrow denotes the nucleotide change.

Through linkage analysis with selected SNPs and microsatellite markers (LOD score equal to minus infinity), 10 ADCC-associated genes were excluded. Five other genes were subject to direct sequencing. These genes were *CRYAA* (D21S1255; LOD score 0.3), *CRYAB* (D11S4151; LOD score 0.9), *CRYGC*,* CRYGD* (D2S2361; LOD score 1.51), and *MAF* (D16S503; LOD score 0.9) in Family A and *CRYAB* (D11S1793; LOD score 2.41), *CRYGC*, and *CRYGD* (D2S2208; LOD score 2.41) in Family B. Two sequence variants in exon 2 of *CRYGD* (NCBI accession number NM_006891.3) were identified to segregate with cataract in these two families. In Family A, a novel missense transversion, c.43C>A, which led to a substitution of arginine with serine at the 15th amino acid position (R15S), was detected (Figure 2). In Family B, a reported missense change, c.70C>A, which substituted proline with threonine at the 24th amino acid position (P24T), was found. All patients showed heterozygous changes. Neither the normal family members nor the 103 unrelated healthy controls carried these changes.

### Computational protein analysis of R15S CRYGD

**Figure 3:**
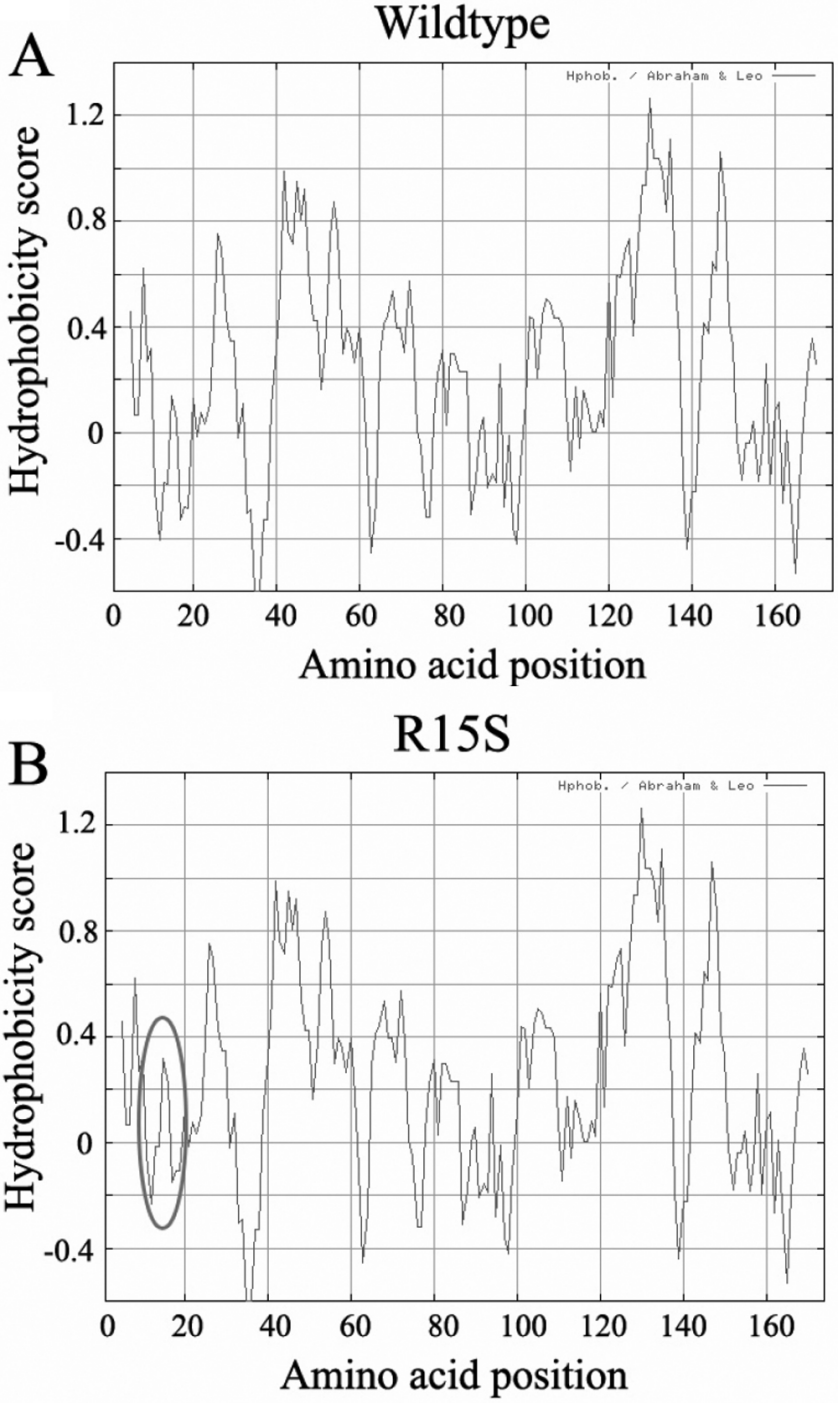
Hydrophobicity change of R15S CRYGD. The prediction by ProtScale analysis at Expasy indicated an increase of local hydrophobicity around the site of R15S mutation (Circle in panel **B**). **A**: The curve showed the hydrophobicity score of each amino acid of wildtype CRYGD. **B**: The curve was the hydrophobicity of R15S CRYGD. At the position of the 15th amino acid and its neighboring locations, the hydrophobicity scores increased which was indicated by the circle.

By Expasy proteomics, R15S CRYGD was predicted to have a reduced isoelectric point (pI) of 6.58 (compared to 7.0 for the wild type). The molecular weight (MW) was also slightly decreased (20.669 kDa for R15S CRYGD versus 20.738 kDa for wild type). By ProtScale analysis, the local hydrophobicity at and near the altered amino acid was increased (Figure 3). By ScanProsite, a hypothetical casein kinase II phosphorylation site was created due to the R15S mutation (Table 4).

**Table 4:** Comparison of wild type and R15S CRYGD amino acid sequences by ScanProsite analysis.

**Hypothetical sites**	**Wild type**	**R15S**
Casein kinase II phosphorylation	5–8: TlyE	5–8: TlyE
		*15–18:ShyE
Protein kinase C phosphorylation	35–37: SaR	35–37: SaR
	75–77: SvR	75–77: SvR
	78–80: ScR	78–80: ScR
	87–89: ShR	87–89: ShR
	166–168: SlR	166–168: SlR
N-glycosylation	50–53: NYSG	50–53: NYSG
Cell attachment sequence	60–62: RGD	60–62: RGD
N-myristoylation	71–76:GLsdSV	71–76:GLsdSV
	158–163:GAtnAR	158–163:GAtnAR
Tyrosine kinase phosphorylation	91–98:Rly,EredY	91–98:Rly,EredY

All predicted functional sites in both wildtype and R15S CRYGD were listed in this table. The number is amino acid position. The asterisk indicates the newly formed casein kinase II phosphorylation site in R15S CRYGD.

### Cell specificity of R15S CRYGD solubility

**Figure 4:**
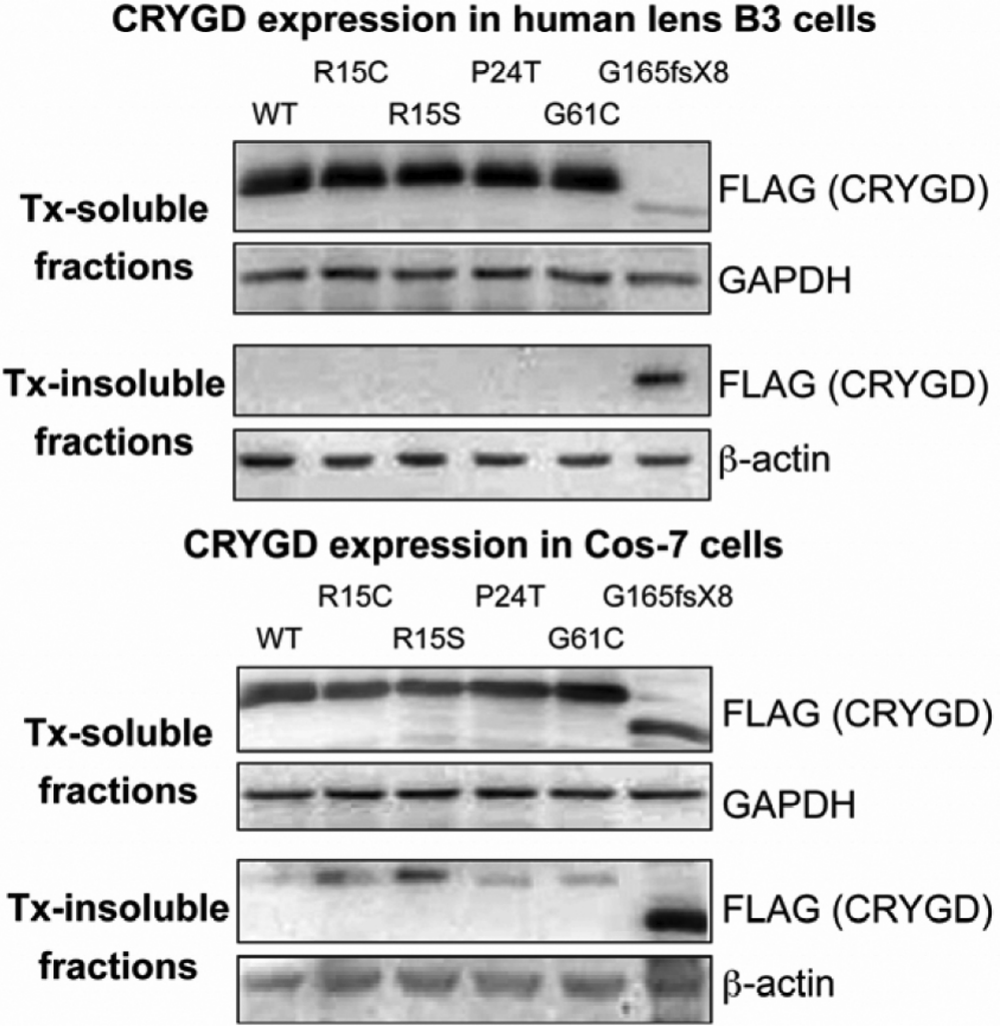
Detergent solubility assay of FLAG-tagged wild type and mutant CRYGD. Western blotting analysis showed cell type specific solubility changes of CRYGD mutants. When expressed in human lens B3 epithelial cells (upper set of blots), wild type (WT) and all known mutant CRYGD (including R15S), except G165fsX8, were completely soluble in 0.5% Triton X-100 extraction. G165fsX8 was mainly Tx-insoluble. When expressed in COS-7 cells (lower set of blots), R15C and R15S CRYGD became moderately Tx-insoluble, unlike the wild type and P24T and G61C mutants. G165fsX8 remained Tx-insoluble.

Recombinant FLAG/myc-tagged wild type or cataract-causing mutant CRYGD (R15S, R15C, P24T, G61C, and G165fsX8) was expressed in human lens epithelial B3 cells and COS-7 cells. Tx-soluble and Tx-insoluble fractions were western blotted for FLAG to detect wild type and mutant CRYGD proteins. The result of expression in lens B3 cells showed that except for G165fsX8, all known mutants of CRYGD remained Tx-soluble, which is similar to the wild type protein (Figure 4). The majority of G165fsX8 was present as Tx-insoluble. However, when expressed in COS-7 cells, an appreciable amount of mutant CRYGD protein became Tx-insoluble (Figure 4) and wild type CRYGD remained Tx-soluble. Band densitometry followed by normalization with housekeeping proteins (GAPDH for Tx-soluble and β-actin for Tx-insoluble fractions) revealed that about 8% of R15C and 17% of R15S CRYGD were Tx-insoluble (compared to 0.2% of wild type CRYGD). Similar observations were obtained in triplicate experiments.

### Cellular distribution

In COS-7 cells, recombinant FLAG/myc-tagged R15S CRYGD was located predominantly in the cytoplasm and slightly in the nuclei (data not shown). No obvious inclusion was observed. This was similar to that observed in cells expressing wild type CRYGD.

## Discussion

In this study, we identified a novel R15S mutation and a P24T change of CRYGD in two Chinese families showing ADCC of the coralliform type, which is a rare subtype of bilateral static congenital cataracts characterized by the appearance of opacity resembling the shape of sea coral. It shows white or cerulean opacification arranged in fusiform or spindle shape, extending from the center of the lens to the periphery but never reaching the capsule [10,11]. The irregular pattern of opacity running across the anatomic boundary of the cortical region indicates an altered arrangement of lens fibers, which associates with light scattering and reduction of transparency [11]. Until now, autosomal dominant transmission was the only mode of inheritance reported for coralliform cataract. Two loci, 2p24-pter and 2q33–35, have been mapped, and the specific gene, *CRYGD* (UniProt), in the latter locus was identified to associate with this cataract subtype [23]. Among all *CRYGD* mutations, R15C, P24T, and G61C were reported to be responsible for the coralliform phenotype [21,24-27]. In this study, the novel R15S change in CRYGD was found to cause congenital coralliform cataract. The index patient, III-10, in in family A did not have a detectable lens opacity when he was 2.5 years old but was diagnosed with cataract at nine years of age. This was different from the effects of a previously reported R15C mutation of CRYGD, which caused the punctate type of congenital cataract at a much earlier age of disease onset [28]. Human CRYGD exists as a monomeric protein with a highly symmetric structure containing four Greek key motifs organized into two highly homologous β-sheet domains. The NH_2_-terminal and COOH-terminal domains are covalently connected by a six-residue linker and interact non-covalently through the side chains of 10 amino acids across the domain interface. Due to these two conserved regions and a central hydrophobic domain interface, CRYGD exhibits high intrinsic stability [29,30]. For the R15C mutant, the additional reactive cysteine molecule at the protein surface could lead to the formation of disulfide cross-linkage, which in turn causes protein aggregation [31]. Nevertheless, substitution of the highly polar and charged arginine molecule by a less polar serine molecule as in R15S may cause lesser effects on protein conformation than the formation of an additional disulfide bond due to cysteine. Therefore, the R15S mutant is associated with the less severe late onset of cataract phenotype in our studied family. Although we detected no alteration of biochemical properties due to R15S in our cell expression studies, increased hydrophobicity at the R15S mutation site as predicted by ProtScale might affect protein–protein interactions since R15 is located on a solvent accessible protein surface [28]. This would contribute to abnormal protein packing in the lens and would result in aggregate formation and thus lens opacification. Moreover, examination by ScanProsite predicted the creation of a putative casein kinase II phosphorylation site due to R15S. Whether this posttranslational modification is associated with protein interactions or oligomerization remains to be investigated. Additionally, an animal study had shown that the mutant γ-crystallin could cause cataract by the deposition of the misfiled protein as amyloid-like inclusions [32]. But this only explained those mutations which could affect protein structure severely, such as a frameshift mutation leading to protein truncation, ultimately causing mutant protein aggregate into inclusion. R15S was not supposed to have such a severe influence on protein structure. No obvious inclusion was observed in cells expressing R15S in this study.

The P24T mutant of CRYGD is associated with diverse opacity morphology [24,25,33-35]. The P24T and P24S mutants [18] are responsible for different crystallin cataracts such as the coralliform, aculeiform, fasciculiform, and crystal forms. In vitro expression of P24T CRYGD demonstrated reduced solubility when compared to the wild type protein [36,37]. The additional formation of a hydrogen bond might modify β-strand conformation, leading to protein insolubility rather than loss of stability. This might result in protein aggregation and then opacification in the lens.

In conclusion, a novel R15S mutation and a P24T mutation of CRYGD were identified in two Chinese families showing the coralliform type of congenital cataract. Different phenotypic features and onset time of disease in the two families accentuated the unique role of a single amino acid change in protein properties, which is crucial for disease pathogenesis. The enhanced hydrophobicity and a hypothetical phosphorylation site in the vicinity of R15S may affect the CRYGD protein interaction and the formation of the protein cluster.

## References

[1] Abrahamsson M Magnusson G Sjostrom A Popovic Z Sjostrand J The occurrence of congenital cataract in western Sweden. Acta Ophthalmol Scand 1999 77 578 80 10551305 10.1034/j.1600-0420.1999.770520.x

[2] Francis PJ Berry V Bhattacharya SS Moore AT The genetics of childhood cataract. J Med Genet 2000 37 481 8 10882749 10.1136/jmg.37.7.481 PMC1734631

[3] Rahi JS Dezateux C Measuring and interpreting the incidence of congenital ocular anomalies: lessons from a national study of congenital cataract in the UK. Invest Ophthalmol Vis Sci 2001 42 1444 8 11381045

[4] Wirth MG Russell-Eggitt IM Craig JE Elder JE Mackey DA Aetiology of congenital and paediatric cataract in an Australian population. Br J Ophthalmol 2002 86 782 6 12084750 10.1136/bjo.86.7.782 PMC1771196

[5] Holmes JM Leske DA Burke JP Hodge DO Birth prevalence of visually significant infantile cataract in a defined U.S. population. Ophthalmic Epidemiol 2003 10 67 74 12660855 10.1076/opep.10.2.67.13894

[6] Haargaard B Wohlfahrt J Fledelius HC Rosenberg T Melbye M Incidence and cumulative risk of childhood cataract in a cohort of 2.6 million Danish children. Invest Ophthalmol Vis Sci 2004 45 1316 20 15111583 10.1167/iovs.03-0635

[7] Reddy MA Francis PJ Berry V Bhattacharya SS Moore AT Molecular genetic basis of inherited cataract and associated phenotypes. Surv Ophthalmol 2004 49 300 15 15110667 10.1016/j.survophthal.2004.02.013

[8] Mackey DA Gregg Lecture: Congenital cataract–from rubella to genetics. Clin Experiment Ophthalmol 2006 34 199 207 16671898 10.1111/j.1442-9071.2006.01194.x

[9] Hejtmancik JF Congenital cataracts and their molecular genetics. Semin Cell Dev Biol 2008 19 134 49 18035564 10.1016/j.semcdb.2007.10.003 PMC2288487

[10] Ionides A Francis P Berry V Mackay D Bhattacharya S Shiels A Moore A Clinical and genetic heterogeneity in autosomal dominant cataract. Br J Ophthalmol 1999 83 802 8 10381667 10.1136/bjo.83.7.802 PMC1723116

[11] Amaya L Taylor D Russell-Eggitt IM The morphology and natural history of childhood cataracts. Surv Ophthalmol 2003 48 125 44 12686301 10.1016/s0039-6257(02)00462-9

[12] Shiels A Hejtmancik JF Genetic origins of cataract. Arch Ophthalmol 2007 125 165 73 17296892 10.1001/archopht.125.2.165

[13] Jamieson RV Farrar N Stewart K Perveen R Mihelec M Carette M Grigg JR McAvoy JW Lovicu FJ Tam PP Scambler P Lloyd IC Donnai D Black GC Characterization of a familial t(16;22) balanced translocation associated with congenital cataract leads to identification of a novel gene, TMEM114, expressed in the lens and disrupted by the translocation. Hum Mutat 2007 28 968 77 17492639 10.1002/humu.20545

[14] Ramachandran RD Perumalsamy V Hejtmancik JF Autosomal recessive juvenile onset cataract associated with mutation in BFSP1. Hum Genet 2007 121 475 82 17225135 10.1007/s00439-006-0319-6

[15] Shiels A Bennett TM Knopf HL CHMP4B, a novel gene for autosomal dominant cataracts linked to chromosome 20q. Am J Hum Genet 2007 81 596 606 17701905 10.1086/519980 PMC1950844

[16] Pras E Raz J Yahalom V Frydman M Garzozi HJ Pras E Hejtmancik JF A nonsense mutation in the glucosaminyl (N-acetyl) transferase 2 gene (GCNT2): association with autosomal recessive congenital cataracts. Invest Ophthalmol Vis Sci 2004 45 1940 5 15161861 10.1167/iovs.03-1117

[17] Hansen L Yao W Eiberg H Kjaer KW Baggesen K Hejtmancik JF Rosenberg T Genetic heterogeneity in microcornea-cataract: five novel mutations in CRYAA, CRYGD, and GJA8. Invest Ophthalmol Vis Sci 2007 48 3937 44 17724170 10.1167/iovs.07-0013

[18] Plotnikova OV Kondrashov FA Vlasov PK Grigorenko AP Ginter EK Rogaev EI Conversion and compensatory evolution of the gamma-crystallin genes and identification of a cataractogenic mutation that reverses the sequence of the human CRYGD gene to an ancestral state. Am J Hum Genet 2007 81 32 43 17564961 10.1086/518616 PMC1950927

[19] Zhang LY Yam GH Fan DS Tam PO Lam DS Pang CP A novel deletion variant of gammaD-crystallin responsible for congenital nuclear cataract. Mol Vis 2007 13 2096 104 18079686

[20] Devi RR Yao W Vijayalakshmi P Sergeev YV Sundaresan P Hejtmancik JF Crystallin gene mutations in Indian families with inherited pediatric cataract. Mol Vis 2008 14 1157 70 18587492 PMC2435160

[21] Li F Wang S Gao C Liu S Zhao B Zhang M Huang S Zhu S Ma X Mutation G61C in the CRYGD gene causing autosomal dominant congenital coralliform cataracts. Mol Vis 2008 14 378 86 18334953 PMC2268897

[22] Yam GHF Gaplovska-Kysela K Zuber Ch Roth J Sodium 4-phenylbutyrate Acts as Chemical Chaperone on Misfolded Myocilin to Rescue Cells from Endoplasmic Reticulum Stress and Apoptosis. Invest Ophthalmol Vis Sci 2007 48 1683 90 17389500 10.1167/iovs.06-0943

[23] Gao L Qin W Cui H Feng G Liu P Gao W Ma L Li P He L Fu S A novel locus of coralliform cataract mapped to chromosome 2p24-pter. J Hum Genet 2005 50 305 10 15933805 10.1007/s10038-005-0251-y

[24] Mackay DS Andley UP Shiels A A missense mutation in the gammaD crystallin gene (CRYGD) associated with autosomal dominant “coral-like” cataract linked to chromosome 2q. Mol Vis 2004 10 155 62 15041957

[25] Shentu X Yao K Xu W Zheng S Hu S Gong X Special fasciculiform cataract caused by a mutation in the gammaD-crystallin gene. Mol Vis 2004 10 233 9 15064679

[26] Xu WZ Zheng S Xu SJ Huang W Yao K Zhang SZ Autosomal dominant coralliform cataract related to a missense mutation of the gammaD-crystallin gene. Chin Med J (Engl) 2004 117 727 32 15161542

[27] Gu F Li R Ma XX Shi LS Huang SZ Ma X A missense mutation in the gammaD-crystallin gene CRYGD associated with autosomal dominant congenital cataract in a Chinese family. Mol Vis 2006 12 26 31 16446699

[28] Stephan DA Gillanders E Vanderveen D Freas-Lutz D Wistow G Baxevanis AD Robbins CM VanAuken A Quesenberry MI Bailey-Wilson J Juo SH Trent JM Smith L Brownstein MJ Progressive juvenile-onset punctate cataracts caused by mutation of the gammaD-crystallin gene. Proc Natl Acad Sci USA 1999 96 1008 12 9927684 10.1073/pnas.96.3.1008 PMC15341

[29] Flaugh SL Kosinski-Collins MS King J Contributions of hydrophobic domain interface interactions to the folding and stability of human gammaD-crystallin. Protein Sci 2005 14 569 81 15722442 10.1110/ps.041111405 PMC2279286

[30] Flaugh SL Kosinski-Collins MS King J Interdomain side-chain interactions in human gammaD crystallin influencing folding and stability. Protein Sci 2005 14 2030 43 16046626 10.1110/ps.051460505 PMC2279314

[31] Pande A Pande J Asherie N Lomakin A Ogun O King JA Lubsen NH Walton D Benedek GB Molecular basis of a progressive juvenile-onset hereditary cataract. Proc Natl Acad Sci USA 2000 97 1993 8 10688888 10.1073/pnas.040554397 PMC15742

[32] Sandilands A Hutcheson AM Long HA Prescott AR Vrensen G Löster J Klopp N Lutz RB Graw J Masaki S Dobson CM MacPhee CE Quinlan RA Altered aggregation properties of mutant gamma-crystallins cause inherited cataract. EMBO J 2002 21 6005 14 12426373 10.1093/emboj/cdf609 PMC137201

[33] Santhiya ST Shyam Manohar M Rawlley D Vijayalakshmi P Namperumalsamy P Gopinath PM Loster J Graw J Novel mutations in the gamma-crystallin genes cause autosomal dominant congenital cataracts. J Med Genet 2002 39 352 8 12011157 10.1136/jmg.39.5.352 PMC1735119

[34] Nandrot E Slingsby C Basak A Cherif-Chefchaouni M Benazzouz B Hajaji Y Boutayeb S Gribouval O Arbogast L Berraho A Abitbol M Hilal L Gamma-D crystallin gene (CRYGD) mutation causes autosomal dominant congenital cerulean cataracts. J Med Genet 2003 40 262 7 12676897 10.1136/jmg.40.4.262 PMC1735438

[35] Burdon KP Wirth MG Mackey DA Russell-Eggitt IM Craig JE Elder JE Dickinson JL Sale MM Investigation of crystallin genes in familial cataract, and report of two disease associated mutations. Br J Ophthalmol 2004 88 79 83 14693780 10.1136/bjo.88.1.79 PMC1771940

[36] Evans P Wyatt K Wistow GJ Bateman OA Wallace BA Slingsby C The P23T cataract mutation causes loss of solubility of folded gammaD-crystallin. J Mol Biol 2004 343 435 44 15451671 10.1016/j.jmb.2004.08.050

[37] Pande A Annunziata O Asherie N Ogun O Benedek GB Pande J Decrease in protein solubility and cataract formation caused by the Pro23 to Thr mutation in human gamma D-crystallin. Biochemistry 2005 44 2491 500 15709761 10.1021/bi0479611

